# Depressed mood, suicidal behaviors, and health risk behaviors among youths in the Commonwealth of the Northern Mariana Islands: the 2017 CNMI Youth Risk Behavior Survey

**DOI:** 10.1186/s12889-020-08663-z

**Published:** 2020-04-15

**Authors:** Jennifer Lisa Sakamoto, Akira Shibanuma, Masamine Jimba

**Affiliations:** Department of Community and Global Health, Graduate School of Medicine, The University of Tokyo, Tokyo, Japan

**Keywords:** Depressed mood, Suicidal behavior, Risk behavior, Adolescent, CNMI, Pacific Island

## Abstract

**Background:**

The current study investigated the prevalence of depressed mood, suicide ideation, suicide plan, and suicide attempt and their associations with health risk behaviors among high school adolescents in the Commonwealth of the Northern Mariana Islands (CNMI).

**Methods:**

This is a cross-sectional study analyzing self-reported data from the 2017 CNMI Youth Risk Behavior Survey (*n* = 1943). Modified Poisson regression models were used to identify the associations between 17 health risk behavior variables, including violence-related behaviors, substance use behaviors, sexual behaviors, and early risk-taking behaviors, and four variables related to depressed mood and suicidal behaviors.

**Results:**

40.7% adolescents reported being depressed, 25.0% reported suicide ideation, 22.8% reported formulating a suicide plan, and 13.6% attempted suicide. Female adolescents were more likely to report depressed mood and all included suicidal behaviors (*p* < 0.001). Being in a physical fight and forced sexual intercourse were associated with depressed mood, suicide ideation, suicide plan, and suicide attempt for both female and male adolescents. Use of “soft drugs” such as current smoking was associated with depressed mood (ARR = 2.33, 95% CI = 1.56–3.45, *p* < 0.001), suicide ideation (ARR = 1.23, 95% CI = 1.08–1.43, *p* < 0.001), suicide plan (ARR = 1.19; 95% CI = 1.05–1.35; *p* < 0.001), and suicide attempt (ARR = 1.18; 95% CI = 1.06–1.30; *p* < 0.001) for females, whereas use of “hard drugs” such as heroin was associated with depressed mood (ARR = 2.27, 95% CI = 1.37–3.85, *p* < 0.01), suicide ideation (ARR = 1.30, 95% CI = 1.01–1.67, *p* < 0.05), suicide plan (ARR = 1.82; 95% CI = 1.22–2.70; *p* < 0.01), and suicide attempt (ARR = 2.78; 95% CI = 1.47–5.26; *p* < 0.01) for male adolescents.

**Conclusion:**

The prevalence of depressed mood, suicide ideation, suicide plan, and suicide attempt among CNMI adolescents was high, especially in female adolescents. While there were gender differences, many of the health risk behaviors were associated with depressed mood and suicidal behaviors. As sociodemographic factors are difficult to change, modifiable factors should be targeted to improve the mental health of adolescents.

## Background

Suicide among adolescents is a major public health concern worldwide. In the US, suicide is the second leading cause of death among adolescents aged 10 to 24 years [1]. Approximately 6,000 adolescents died by suicide in 2017, and many more seriously considered and attempted suicide [2]. Those who experience a suicidal crisis typically engage in a continuum of suicidal behaviors from seriously considering suicide (suicide ideation), formulating plans of suicide (suicide plan), and carrying out suicidal acts (suicide attempt) to death by suicide [3–5]. Many people who have ever considered or attempted suicide did so for the first time during their youth, as the lifetime age of onset for suicidal ideation and suicide attempt typically occurs during adolescence to early adulthood [6]. Moreover, depressive symptoms are strongly associated with suicidal behaviors, and experiencing depression during adolescence can lead to severe adverse outcomes later in adulthood [3, 5, 7].

Suicide deaths among adolescents are preventable by overcoming risk factors such as bullying [7], victimization [7], substance abuse [8], and sexual activity [9]. Because suicide is often a complex interaction of psychological, social, biological, cultural, and environmental factors [10, 11], it is crucial to identify the risk factors from various aspects of an adolescent’s life.

In the Micronesia region encompassing the former US Trust Territories of the Pacific Islands—which include the three inhabited unincorporated island territories of Guam, the Commonwealth of the Northern Mariana Islands (CNMI), and American Samoa—the adolescent suicide rate is among the highest in the world [12, 13]. Specifically, the suicide rate among male adolescents is high in this region, and prior research has explained this epidemiological finding [14]. In the past two centuries, US territories of the Pacific have faced major sociocultural changes through the colonial rule of Spain, Germany, Japan, and finally the US. After World War II until the mid-1960s, rapid cultural changes occurred as westernization greatly influenced society through the introduction of American-style schools and health services and modern forms of technology. Economic changes accompanied these sociocultural changes. The islands shifted from a subsistence economy relying on fishing and farming to a cash economy and widespread government employment [15].

Alongside this major economic transformation, fundamental changes in interpersonal relationships were simultaneously occurring, which led to intergenerational conflict and isolation among adolescents in this region [16]. More recently, a study conducted in Guam suggests that unemployment is particularly high among male adolescents in the US territories of the Pacific because they often lack the skills required for private-sector employment and other high-level jobs. This results in loss of social connections related to employment, resulting in further isolation from society [17]. Although the high suicide rates in the Micronesian region, including the US territories of the Pacific, have drawn attention, most related research has focused primarily on male adolescents and suicide deaths. Previous research has explored suicidal behaviors among US high school adolescents through an analysis of the Youth Risk Behavior Survey (YRBS) [5, 18], a school-based survey conducted by the Centers for Disease Control and Prevention (CDC). Through this survey, the Centers monitor six categories of health-related behaviors—behaviors that lead to unintentional injuries and violence, risky sexual behaviors, alcohol and other drug use, unhealthy dietary behaviors, and inadequate physical activity, all of which contribute to mortality and morbidity among adolescents. However, all US territories, including the CNMI, were excluded from the national YRBS data during the sample selection process [19]. Due to the distinct history and culture of the indigenous population in the US territories of the Pacific [20], the results of the CNMI YRBS might differ from the US national YRBS results.

The current study was conducted to investigate the prevalence of depressed mood and suicidal behaviors and their association with health risk behaviors among high school adolescents in the CNMI.

## Methods

### Data source and participants

This is a cross-sectional study analyzing data from the 2017 CNMI YRBS. The YRBS is sponsored by the CDC and is conducted biennially to monitor health risk behaviors that contribute to the leading causes of death, disability, and social problems among adolescents and adults in the US. The CNMI Public School System conducted the 2017 CNMI YRBS among 9th to 12th graders in CNMI public schools using a two-stage cluster sample design to produce an island-wide representative sample of students. Survey procedures were designed to protect adolescents’ privacy through anonymity and encourage voluntary participation. Before administering the survey, local parental permission procedures were followed; these included handing out parental permission forms to the students prior to the survey, and parents who did not want their children to participate in the survey could return the permission forms to the teachers. Adolescents whose parents granted permission to participate completed the self-administered questionnaire on a computer-scannable answer sheet. Because the data used in these analyses contain no personally identifiable information, this study was exempt from institutional board review. Among the three US Pacific territories (Guam, Puerto Rico, CNMI) that conducted the YRBS in 2017, CNMI had the largest sample size of 1943 adolescents and an overall response rate of 64%. A weight was applied to each record to adjust for student nonresponse and equitable distribution of students by grade and gender.

In 2017, the national YRBS questionnaire included 99 questions. The 2017 CNMI YRBS included 89 questions from the national YRBS questionnaire that were relevant to its cultural context.

### Depressed mood and suicidal behaviors

The outcome variables were depressed mood and suicidal behaviors, which were measured by four questions in the CNMI YRBS under behaviors related to violence: depressed mood, suicide ideation, suicide plan, and suicide attempt. The test re-test conducted by the CDC to measure the reliability of the four questions showed moderate to substantial reliability [21]. Specifically, kappa values of 56.4% for depressed mood, 74.3% for suicide ideation, 66.6% for suicide plan, and 72.7% for suicide attempt were obtained [22]. Another study measuring the validity of suicidality items in the YRBS found that these items are valid and thus useful for assessing suicidal behaviors [23]. *Depressed mood* was assessed with the question: “During the past 12 months, did you ever feel so sad or hopeless almost every day for two weeks or more in a row that you stopped doing some usual activities?” *Suicide ideation* was assessed with the question: “During the past 12 months, did you ever seriously consider attempting suicide?” *Suicide plan* was assessed with the question: “During the past 12 months, did you make a plan about how you would attempt suicide?” *Suicide attempt* was assessed with the question: “During the past 12 months, how many times did you actually attempt suicide?”

### Health risk behaviors

Health risk behaviors were measured by 17 independent variables and classified into four domains: violence-related behaviors, substance use behaviors, sexual behaviors, and early risk-taking behaviors. The questions and response choices are specified in Table 1.

**Table 1:** Questions on depressed mood, suicidal behaviors, and health risk behaviors with response choices

	Questions	Response choices
Depressed mood		
Depressed mood	During the past 12 months, did you ever feel so sad or hopeless almost every day for two weeks or more in a row that you stopped doing some usual activities?	1: Yes; 2: No
Suicidal behaviors		
Suicide ideation	During the past 12 months, did you ever seriously consider attempting suicide?	1: Yes; 2: No
Suicide plan	During the past 12 months, did you make a plan about how you would attempt suicide?	1: Yes; 2: No
Suicide attempt	During the past 12 months, how many times did you actually attempt suicide?	1: 0 times; 2: 1 + time
Violence		
Carried a weapon	During the past 30 days, on how many days did you carry a weapon such as gun, knife, or club?	1: 0 days; 2: 1 + day
Physical fight	During the past 12 months, how many times were you in a physical fight?	1: 0 times; 2: 1 + time
Forced sexual intercourse	Have you ever been physically forced to have sexual intercourse when you did not want to?	1: Yes; 2: No
Physical dating violence	During the past 12 months, how many times did someone you were dating or going out with physically hurt you on purpose?	1: I did not date or go out with anyone during the past 12 months or 0 times; 2: 1 + time
Bullied on school property	During the past 12 months, have you ever been bullied on school property?	1: Yes; 2: No
Substance use		
Current smoking	During the past 30 days, on how many days did you smoke cigarettes?	1: 0 days; 2: 1 + day
Current alcohol use	During the past 30 days, on how many days did you have at least one drink of alcohol?	1: 0 days; 2: 1 + day
Current marijuana use	During the past 30 days, how many times did you use marijuana on school property?	1: 0 times; 2: 1 + time
Ever cocaine use	During your life, how many times have you used any form of cocaine, including powder, crack, or freebase?	1: 0 times; 2: 1 + time
Ever heroin use	During your life, how many times have you used heroin (also called smack, junk, or China White)?	1: 0 times; 2: 1 + time
Ever methamphetamine use	During your life, how many times have you used methamphetamines (also called speed, crystal, crank, or ice)?	1: 0 times; 2: 1 + time
Sexual behavior		
Currently sexually active	During the past 3 months, how many people have you had sexual intercourse with?	1: I did not date or go out with anyone during the past 12 months or 0 person; 2: 1 + people
Sex with 4 or more in life	During your life, with how many people have you had sexual intercourse?	1: I have never had sexual intercourse or 1–3 people; 2: 4 + people
Early risk-taking behavior		
Smoking under age 13	How old were you when you smoked a whole cigarette for the first time?	1: I have never smoked or 13 years old+; 2: 12 years old or younger
Drinking under age 13	How old were you when you had your first alcohol other than a few sips?	1: I have never had a drink or 13 years old+; 2: 12 years old or younger
Marijuana use under age 13	How old were you when you tried marijuana for the first time?	1: I have never tried marijuana or 13 years old+; 2: 12 years old or younger
Sex under age 13	How old were you when you had sexual intercourse for the first time?	1: I have never had sexual intercourse or 13 years old+; 2: 12 years old or younger

Violence-related behaviors were assessed with five questions regarding: *carrying a weapon*, *physical fight*, *forced sexual intercourse*, *physical dating violence*, and *being bullied on school property*. Substance use behaviors were assessed with six questions regarding: *current smoking*, *current alcohol use*, *current marijuana use*, *ever cocaine use*, *ever heroin use*, and *ever methamphetamine use*. Sexual behaviors were assessed with two questions regarding: *current sexual activity status* and *sex with four or more partners in life*. Early risk-taking behaviors were assessed with four questions regarding: *smoking under age 13*, *drinking under age 13*, *marijuana use under age 13*, and *sex under age 13*.

### Demographics

Demographic information, including age, gender, grade, and race/ethnicity, were all self-reported. Age was reported as one of the following categories: 12 years or younger, 13 years, 14 years, 15 years, 16 years, 17 years, or 18 years or older. Gender was reported as female or male. Grade was reported as one of the following categories: 9th grade, 10th grade, 11th grade, 12th grade, or ungraded/other. Race/ethnicity was reported as one of the following categories: Asian, Native Hawaiian or other Pacific Islander, Black or African American, Hispanic/Latino, White, American Indian/Alaskan Native, Hispanic with Multiple Race/Ethnicity, or Non-Hispanic Multiple Race/Ethnicity.

Academic performance was self-reported and assessed with the question: “During the past 12 months, how would you describe your grades in school?” The response options were Mostly A’s, Mostly B’s, Mostly C’s, Mostly D’s, Mostly F’s, and Not sure.

### Statistical analyses

To account for the complex sample design of the YRBS, all analyses used the complex sample survey procedures in software program Stata version 13 (StataCorp, College Station, TX) using svy commands. Prevalence estimates reported herein reflect weighted estimates. Due to the strongly positively skewed distributions, depressed mood, suicidal behavior, and health risk behavior questions were dichotomized as “yes” or “no.” The alpha was set at *p* < 0.05. A Rao-Scott chi-square test was used to separately assess the association between depressed mood, suicidal behaviors, and demographic variables. Modified Poisson regression models were used to separately assess the association of each health risk behavior variable with each of the following outcomes: depressed mood, suicide ideation, suicide plan, and suicide attempt [24, 25]. Adjusted relative risks (adjusting for age, race/ethnicity, and academic performance) along with 95% confidence intervals were reported. Multicollinearity was tested using tolerance and the variance inflation factor; as a result, grade was excluded but age was retained in all adjusted models.

## Results

### Demographic characteristics, academic performance, and health risk behaviors of adolescents

A total of 1943 high school adolescents took the 2017 CNMI YRBS (Table 2). Among these, 49.8% identified as female adolescents. Most adolescents identified as Native Hawaiian or Pacific Islander (46.5%), followed by Asian (41.1%). Academic performance was self-reported, and most reported receiving A’s (33.1%) or B’s (32.1%). Because of the small sample size, adolescents aged 12 years or younger, 13 years, and 14 years were combined into one category.

**Table 2:** Demographic variables and academic performance of adolescents

	Total (***n*** = 1943)	
	Unweighted Frequency (n)	Weighted Frequency (%)
**Demographic variables**		
Age		
<=14 years old	207	9.6
15 years old	536	25.2
16 years old	547	28.5
17 years old	453	25.7
> = 18 years old	199	11.0
Missing	1	
Gender		
Female	951	49.8
Male	982	50.2
Missing	10	
Grade		
9th grade	623	29.3
10th grade	557	26.8
11th grade	502	31.1
12th grade	242	12.7
Ungraded or other grade	5	0.3
Missing	14	
Race/Ethnicity		
Asian	762	41.1
Pacific Islander	874	46.5
Black	11	0.6
Hispanic	2	0.1
White	12	0.7
American Indian/Alaskan Native	2	0.1
Multiple-Hispanic/Latino	103	5.6
Multiple-Non Hispanic/Latino	100	5.3
Missing	77	
**Academic performance**		
Mostly A’s	604	33.0
Mostly B’s	593	32.2
Mostly C’s	314	17.2
Mostly D’s	125	6.7
Mostly F’s	31	1.4
None of these grades	5	0.3
Not sure	174	9.3
Missing	97	

Table 3 shows the prevalence of health risk behaviors among the adolescents in unweighted frequencies and weighted percentages. Among the adolescents, 502 (26.0%) reported using marijuana at least one or more times in the past 30 days and 393 (24.3%) reported being currently sexually active.

**Table 3:** Prevalence of health risk behaviors among adolescents

	Total (***n*** = 1943)	
Health risk behaviors	Unweighted Frequency (n)	Weighted Frequency (%)
**Violence-related behaviors**		
Carried a weapon	264	13.0
Physical fight	410	20.9
Forced sexual intercourse	245	13.1
Physical dating violence	94	9.1
Bullied on school property	464	23.2
**Substance use behaviors**		
Current smoking	231	12.4
Current alcohol use	381	23.3
Current marijuana use	502	26.0
Ever use of cocaine	70	3.6
Ever use of heroin	56	3.0
Ever use of methamphetamine	59	3.4
**Sexual behaviors**		
Currently sexually active	393	24.3
Sex with 4 or more in life	99	5.9
**Early risk-taking behaviors**		
Smoking under age 13	328	17.4
Drinking under age 13	336	18.7
Marijuana use under age 13	239	12.5
Sex under age 13	69	4.1

### Prevalence of depressed mood and suicidal behaviors

Overall, 786 (40.7%) adolescents reported that they were in a depressed mood every day for two or more weeks in a row, causing them to stop doing some usual activities during the 12 months before the survey; in addition, 498 (25.0%) adolescents reported suicide ideation, 459 (22.8%) reported formulating a suicide plan, and 241 (13.6%) attempted suicide during the past 12 months. As Table 4 shows, female adolescents were significantly more likely to report depressed mood and all included suicidal behaviors than male adolescents. Differences by grade were not observed in depressed mood and suicidal behaviors (Table 4).

**Table 4:** Prevalence of depressed mood and suicidal behaviors by demographic variables and academic performance

Demographic variables	Depressed mood		Suicide ideation		Suicide plan		Suicide attempt	
	Yes (***n*** = 786)	***p***-value	Yes (***n*** = 498)	***p***-value	Yes (***n*** = 459)	***p***-value	Yes (***n*** = 241)	***p***-value
Gender		**< 0.0001**		**< 0.0001**		**< 0.0001**		**< 0.0001**
Female	49.5		32.5		28.4		17.2	
Male	32.0		17.5		17.2		9.8	
Age		0.211		0.286		0.325		**0.047**
<=14 years old	9.4		8.8		10.7		10.9	
15 years old	26.8		25.6		26.3		28.4	
16 years old	30.1		31.6		31.5		31.7	
17 years old	24.6		22.7		23.3		21.5	
> = 18 years old	9.1		11.3		8.3		7.5	
Grade		0.062		0.809		0.536		0.446
9th grade	45.0		25.5		25.0		15.0	
10th grade	40.2		23.9		23.7		14.4	
11th grade	40.5		24.6		21.3		12.7	
12th grade	33.0		27.0		19.4		8.8	
Race/Ethnicity		0.137		**0.041**		0.474		**0.045**
Asian	39.6		21.8		20.9		10.0	
Pacific Islander	41.3		28.5		24.9		15.5	
Black	21.3		33.2		19.9		16.6	
Hispanic	44.2		0.0		0.0		0.0	
White	11.5		0.0		16.2		12.4	
Academic performance		**0.042**		**0.001**		**0.002**		**0.001**
Mostly A’s	35.0		22.3		19.3		10.3	
Mostly B’s	41.8		21.7		21.8		11.2	
Mostly C’s	41.1		27.7		23.2		14.9	
Mostly D’s	47.9		27.1		25.7		16.1	
Mostly F’s	58.8		57.8		54.9		32.5	

Note. Rao-Scott chi-square test was used to test the independence regarding the combination of a demographic or academic performance variable and an outcome

### Violence-related behaviors associated with depressed mood and suicidal behaviors

Table 5 and Table 6 show the adjusted relative risk (ARR) and unadjusted relative risk (RR) of health risk behaviors associated with depressed mood and suicidal behaviors. Being involved in a physical fight and forced sexual intercourse were positively associated with depressed mood, suicide ideation, suicide plan, and suicide attempt for both male and female adolescents. Carrying a weapon was associated with suicide ideation (ARR = 1.12, 95% confidence interval (CI) = 1.01–1.23, *p* < 0.05) and suicide attempt (ARR = 1.10, 95% CI = 1.01–1.20, *p* < 0.05) only for males. Experiencing bullying on school property was positively associated with depressed mood (ARR = 1.59, 95% CI = 1.28–1.96, *p* < 0.001), suicide ideation (ARR = 1.39, 95% CI = 1.19–1.61, *p* < 0.001), suicide plan (ARR = 1.33, 95% CI = 1.16–1.52, *p* < 0.001), and suicide attempt (ARR = 1.27, 95% CI = 1.14–1.39, *p* < 0.001) for female adolescents.

**Table 5:** Association between depressed mood, suicidal behaviors, and health risk behaviors of adolescents by gender, adjusted relative risk

	Depressed mood		Suicide ideation		Suicide plan		Suicide attempt	
Health risk behaviors	Female (*n* = 472), ARR (95% CI)	Male (*n* = 332), ARR (95% CI)	Female (*n* = 347), ARR (95% CI)	Male (*n* = 195), ARR (95% CI)	Female (*n* = 336), ARR (95% CI)	Male (*n* = 218), ARR (95% CI)	Female (*n* = 193), ARR (95% CI)	Male (*n* = 92), ARR (95% CI)
**Violence**								
Carried a weapon	1. 49 (0.99, 2.22)	1.08 (0.93, 1.23)	1.19 (0.94, 1.49)	**1.12 (1.01, 1.23)**	1.18 (0.96, 1.45)	1.10 (0.99, 1.20)	1.18 (0.98, 1.41)	**1.10 (1.01, 1.20)**
Physical fight	**1.56 (1.18, 2.08)**	**1.41 (1.20, 1.64)**	**1.32 (1.08, 1.61)**	**1.12 (1.03, 1.23)**	**1.33 (1.15, 1.56)**	**1.20 (1.08, 1.33)**	**1.54 (1.28, 1.89)**	**1.10 (1.03, 1.18)**
Forced sexual intercourse	**2.27 (1.54, 3.33)**	**1.52 (1.15, 2.04)**	**1.54 (1.23, 1.92)**	**1.37 (1.12, 1.67)**	**1.32 (1.12, 1.54)**	**1.33 (1.10, 1.61)**	**1.28 (1.12, 1.47)**	**1.22 (1.04, 1.43)**
Physical dating violence	1.64 (0.88, 3.03)	**1.56 (1.12, 2.13)**	**1.59 (1.04, 2.44)**	**1.79 (1.37, 2.38)**	**1.64 (1.15, 2.33)**	**1.89 (1.33, 2.63)**	**1.75 (1.22, 2.50)**	**1.92 (1.33, 2.78)**
Bullied on school property	**1.59 (1.28, 1.96)**	**1.49 (1.27, 1.82)**	**1.39 (1.19, 1.61)**	**1.23 (1.09, 1.37)**	**1.33 (1.16, 1.52)**	**1.33 (1.19, 1.52)**	**1.27 (1.14, 1.39)**	1.06 (1.00, 1.14)
**Substance use**								
Current smoking	**2.33 (1.56, 3.45)**	1.08 (0.93, 1.23)	**1.64 (1.27, 2.13)**	**1.05 (0.94, 1.18)**	**1.45 (1.16, 1.79)**	1.06 (0.97, 1.16)	**1.59 (1.25, 2.04)**	**1.11 (1.01, 1.20)**
Current alcohol use	**1.54 (1.22, 1.96)**	1.15 (1.00, 1.33)	**1.28 (1.11, 1.47)**	**1.06 (0.96, 1.18)**	**1.27 (1.09, 1.45)**	**1.14 (1.02, 1.27)**	**1.23 (1.08, 1.41)**	**1.08 (1.00, 1.15)**
Current marijuana use	**1.45 (1.18, 1.79)**	1.06 (0.94, 1.22)	**1.23 (1.06, 1.43)**	1.04 (0.96, 1.14)	**1.16 (1.03, 1.33)**	1.01 (0.93, 1.09)	**1.12 (1.01, 1.25)**	1.03 (0.97, 1.10)
Ever cocaine use	0.72 (0.43, 1.19)	**1.41 (1.08, 1.82)**	0.80 (0.54, 1.19)	1.19 (0.93, 1.52)	0.88 (0.59, 1.30)	1.30 (1.00, 1.69)	1.61 (0.68, 3.85)	**1.64 (1.16, 2.33)**
Ever heroin use	0.75 (0.36, 1.56)	**2.17 (1.35, 3.45)**	1.18 (0.57, 2.44)	1.27 (0.96, 1.67)	1.72 (0.67, 4.55)	**1.85 (1.20, 2.86)**	2.33 (0.63, 9.09)	**2.70 (1.32, 5.56)**
Ever methamphetamine use	0.73 (0.44, 1.20)	**1.72 (1.19, 2.50)**	1.18 (0.63, 2.17)	1.16 (0.92, 1.47)	1.39 (0.74, 2.56)	**1.64 (1.18, 2.27)**	2.13 (0.72, 6.25)	**1.79 (1.33, 2.38)**
**Sexual behavior**								
Currently sexually active	1.18 (0.95, 1.47)	1.01 (0.89, 1.15)	**1.23 (1.08, 1.43)**	1.10 (0.99, 1.20)	**1.19 (1.05, 1.35)**	1.05 (0.97, 1.15)	**1.18 (1.06, 1.30**)	1.08 (1.00, 1.16)
Sex with 4 or more in life	1.69 (0.93, 3.13**)**	1.16 (0.93, 1.47)	1.11 (0.83, 1.49)	1.10 (0.89, 1.35)	1.01 (0.81, 1.28)	1.06 (0.92, 1.22)	1.16 (0.93, 1.45)	1.06 (0.97, 1.18)
**Early risk-taking behavior**								
Smoking under age 13	1.12 (0.93, 1.35)	1.08 (0.95, 1.22)	1.18 (0.99, 1.41)	1.05 (0.96, 1.16)	**1.19 (1.02, 1.37)**	**1.14 (1.03, 1.25)**	1.08 (0.95, 1.22)	1.02 (0.95, 1.09)
Drinking under age 13	1.25 (0.98, 1.61)	1.09 (0.94, 1.25)	**1.19 (1.04, 1.39)**	1.09 (0.98, 1.22)	**1.23 (1.08, 1.43)**	**1.14 (1.02, 1.27)**	1.10 (0.99, 1.22)	**1.11 (1.03, 1.20)**
Marijuana use under age 13	0.99 (0.75, 1.32)	**1.16 (1.00, 1.37)**	1.11 (0.88, 1.39)	1.00 (0.89, 1.11)	1.09 (0.92, 1.28)	1.01 (0.91, 1.11)	1.19 (1.00, 1.41)	1.02 (0.95, 1.10)
Sex under age 13	1.37 (0.83, 2.22)	1.08 (0.84, 1.37)	1.19 (0.83, 1.69)	0.98 (0.85, 1.12)	1.09 (0.82, 1.43)	0.97 (0.85, 1.11)	1.01 (0.83, 1.22)	1.00 (0.91, 1.11)

Note. *ARR* adjusted relative risk, *CI* confidence interval. Each ARR and its 95% CI was estimated in a separate model, adjusted for age, race/ethnicity, and academic performance. The numbers in bold indicate significance at *p* < 0.05

**Table 6:** Association between depressed mood, suicidal behaviors, and health risk behaviors of adolescents by gender, unadjusted relative risk

	Depressed mood		Suicide ideation		Suicide plan		Suicide attempt	
Health risk behaviors	Female (n = 472), RR (95% CI)	Male (n = 332), RR (95% CI)	Female (n = 347), RR (95% CI)	Male (n = 195), RR (95% CI)	Female (n = 336), RR (95% CI)	Male (n = 218), RR (95% CI)	Female (n = 193), RR (95% CI)	Male (n = 92), RR (95% CI)
**Violence**								
Carried a weapon	1.49 (0.99, 2.22)	1.15 (0.90, 1.45)	1.32 (0.88, 1.96)	**1.28 (1.03, 1.61)**	**1.45 (1.02, 2.04)**	1.11 (0.93, 1.30)	1.47 (0.88, 2.44)	**1.25 (1.00, 1.56)**
Physical fight	**1.72 (1.32, 2.22)**	**1.35 (1.18, 1.56)**	**1.43 (1.15, 1.79)**	**1.14 (1.05, 1.23)**	**1.45 (1.22, 1.75)**	**1.22 (1.11, 1.33)**	**1.61 (1.35, 1.96)**	**1.14 (1.05, 1.22)**
Forced sexual intercourse	**2.33 (1.67, 3.23)**	**1.45 (1.14, 1.85)**	**1.52 (1.22, 1.85)**	**1.33 (1.12, 1.59)**	**1.32 (1.14, 1.54)**	**1.30 (1.09, 1.54)**	**1.28 (1.15, 1.43)**	**1.30 (1.11, 1.54)**
Physical dating violence	**2.17 (1.22, 3.85)**	**1.64 (1.16, 2.27)**	**1.67 (1.14, 2.50)**	**1.85 (1.41, 2.44)**	**1.72 (1.23, 2.38)**	**1.85 (1.33, 2.56)**	**1.79 (1.25, 2.56)**	**1.96 (1.35, 2.78)**
Bullied on school property	**1.67 (1.37, 2.04)**	**1.56 (1.30, 1.85)**	**1.43 (1.23, 1.64)**	**1.23 (1.10, 1.39)**	**1.35 (1.20, 1.52)**	**1.35 (1.20, 1.54)**	**1.25 (1.14, 1.37)**	1.06 (1.00, 1.12)
**Substance use**								
Current smoking	**2.38 (1.67, 3.45)**	1.08 (0.93, 1.22)	**1.75 (1.37, 2.27)**	1.08 (0.98, 1.19)	**1.54 (1.27, 1.89)**	1.05 (0.97, 1.15)	**1.67 (1.30, 2.17)**	**1.15 (1.06, 1.25)**
Current alcohol use	**1.59 (1.27, 2.00)**	1.14 (0.99, 1.30)	**1.30 (1.12, 1.49)**	1.09 (0.99, 1.19)	**1.28 (1.11, 1.47)**	**1.11 (1.01, 1.22)**	**1.25 (1.09, 1.43)**	**1.10 (1.02, 1.19)**
Current marijuana use	**1.47 (1.20, 1.82)**	1.05 (0.93, 1.18)	**1.25 (1.11, 1.43)**	1.09 (1.00, 1.18)	**1.19 (1.08, 1.32)**	1.01 (0.94, 1.09)	**1.14 (1.03, 1.25)**	1.04 (0.98, 1.10)
Ever cocaine use	0.92 (0.51, 1.64)	**1.39 (1.02, 1.89)**	0.80 (0.62, 1.47)	1.19 (0.96, 1.47)	1.01 (0.62, 1.61)	1.27 (0.97, 1.64)	1.67 (0.72, 3.85)	**1.75 (1.23, 2.50)**
Ever heroin use	1.06 (0.52, 2.17)	**2.27 (1.37, 3.85)**	1.22 (0.68, 2.17)	**1.30 (1.01, 1.67)**	1.61 (0.76, 3.45)	**1.82 (1.22, 2.70)**	1.79 (0.72, 4.35)	**2.78 (1.47, 5.26)**
Ever methamphetamine use	0.83 (0.52, 1.32)	**1.69 (1.12, 2.50)**	1.28 (0.67, 2.44)	1.19 (0.95, 1.52)	1.49 (0.78, 2.86)	**1.56 (1.16, 2.13)**	2.13 (0.70, 6.67)	**2.04 (1.47, 2.78)**
**Sexual behavior**								
Currently sexually active	**1.25 (1.02, 1.52)**	1.01 (0.89, 1.14)	**1.27 (1.10, 1.47)**	**1.10 (1.01, 1.20)**	**1.20 (1.06, 1.35)**	1.04 (0.96, 1.14)	**1.20 (1.08, 1.33)**	**1.10 (1.01, 1.19)**
Sex with 4 or more in life	**1.89 (1.00, 3.45)**	1.20 (0.95, 1.54)	1.15 (0.85, 1.54)	1.12 (0.93, 1.35)	1.04 (0.82, 1.32)	1.05 (0.92, 1.20)	1.20 (0.95, 1.54)	1.18 (1.03, 1.35)
**Early risk-taking behavior**								
Smoking under age 13	**1.20 (1.01, 1.43)**	1.04 (0.93, 1.18)	**1.23 (1.04, 1.47)**	1.09 (1.00, 1.19)	**1.22 (1.05, 1.43)**	**1.12 (1.03, 1.23)**	1.10 (0.98, 1.23)	1.04 (0.97, 1.12)
Drinking under age 13	**1.33 (1.05, 1.69)**	1.08 (0.93, 1.23)	**1.23 (1.08, 1.41)**	1.11 (1.00, 1.23)	**1.27 (1.10, 1.45)**	**1.14 (1.03, 1.25)**	**1.11 (1.00, 1.22)**	**1.10 (1.02, 1.18)**
Marijuana use under age 13	1.14 (0.87, 1.49)	1.10 (0.96, 1.27)	1.19 (0.98, 1.45)	1.02 (0.93, 1.12)	1.16 (0.98, 1.37)	1.01 (0.91, 1.11)	1.27 (1.08, 1.52)	1.09 (1.00, 1.19)
Sex under age 13	1.39 (0.80, 2.38)	1.06 (0.84, 1.33)	1.20 (0.84, 1.72)	1.02 (0.88, 1.19)	1.09 (0.81, 1.43)	0.97 (0.85, 1.09)	1.02 (0.84, 1.23)	1.05 (0.92, 1.22)

Note. *RR* relative risk, *CI* = confidence interval. The numbers in bold indicate significance at *p* < 0.05

### Substance use behaviors associated with depressed mood and suicidal behaviors

Being a current smoker, current alcohol user, and current marijuana user were associated with depressed mood, suicide ideation, suicide plan, and suicide attempt exclusively for female adolescents. Among male adolescents, ever use of heroin (depressed mood: ARR = 2.17, 95% CI = 1.35–3.45, *p* < 0.01; suicide plan: ARR = 1.85, 95% CI = 1.20–2.86, *p* < 0.01; suicide attempt: ARR = 2.70, 95%CI = 1.32–5.56, *p* < 0.01) and methamphetamine (depressed mood: ARR = 1.72, 95% CI = 1.19–2.50, *p* < 0.01; suicide plan: ARR = 1.64, 95% CI = 1.18–2.27, *p* < 0.01; suicide attempt: ARR = 1.79, 95%CI = 1.33–2.38, *p* < 0.001) were associated with depressed mood, suicide plan, and suicide attempt, while none of these were associated among females.

### Sexual behaviors associated with depressed mood and suicidal behaviors

Regarding sexual behaviors, being currently sexually active was associated with suicide ideation (ARR = 1.23, 95% CI = 1.08–1.43, *p* < 0.01), suicide plan (ARR = 1.19, 95% CI = 1.05–1.35, *p* < 0.01), and suicide attempt (ARR = 1.18, 95% CI = 1.06–1.30, *p* < 0.01) for female adolescents.

### Early risk-taking behaviors associated with depressed mood and suicidal behaviors

Smoking before the age of 13 was positively associated with suicide plan for both female and male adolescents. Drinking alcohol before the age of 13 was positively associated with suicide plan (ARR = 1.14, 95% CI = 1.02–1.27, *p* < 0.05) and suicide attempt (ARR = 1.11, 95% CI = 1.03–1.20, *p* < 0.01) for male adolescents.

## Discussion

The prevalence of depressed mood and suicidal behaviors among CNMI high school adolescents was higher than their national prevalence in the US. Although male adolescents have a higher suicide rate in the US Trust Territories of the Pacific Islands [15], female adolescents are more likely to experience depressed mood and suicidal behaviors. The current study extends the finding that adolescents experiencing depressed mood and suicidal behaviors often engage in health risk behaviors. Specifically, use of “soft drugs” such as alcohol was associated with depressed mood, suicide ideation, suicide plan, and suicide attempt among female adolescents, whereas use of “hard drugs” such as cocaine showed greater associations among male adolescents.

In the 2017 CNMI YRBS, 40.7% of adolescents reported being depressed, 25.0% reported suicide ideation, 22.8% reported formulating a suicide plan, and 13.6% attempted suicide. By contrast, in the 2017 national YRBS [22], 31.5% reported being depressed, 17.2% reported suicide ideation, 13.6% reported formulating a suicide plan, and 7.4% attempted suicide. Thus, CNMI adolescents were more likely to experience depressed mood and suicidal behaviors than adolescents in the US mainland. Because more than half of the adolescents in this study were Pacific Islanders, including the indigenous population of the CNMI, distinct cultural factors may have affected these results [20]. Indigenous people commonly experience social and economic marginalization and consequent disparities in health [26]. In the US Census, CNMI had a poverty rate of 55.7% [27], while the national poverty rate was 12.3% [28]. These data suggest that economic barriers might prevent adolescents in the CNMI from accessing and receiving proper mental health care, such as counseling and therapy. Further research is also necessary to investigate the fear and stigmatization surrounding mental health in the Pacific Islands, as these may have acted as social barriers to adolescents consulting mental health professionals [29].

In this study, female adolescents were more likely to experience depressed mood, suicide ideation, suicide plan, and suicide attempt than male adolescents in the CNMI, perhaps because of transitioning gender roles. Traditionally, Micronesian societies were matrilineal, granting women important roles in the community and equal power as men. However, due to societal transition, the culture is shifting to patrilineality, increasing the economic and social marginalization of women [16]. One study implicated the low status of women as an important indicator of suicide in the Pacific Islands [13]. These societal transitions can cause female adolescents to be more vulnerable in society, which can possibly lead to depressed mood and suicidal behaviors.

Being in a physical fight and forced sexual intercourse were associated with depressed mood, suicide ideation, suicide plan, and suicide attempt. In Micronesian culture, including the CNMI, one of the major precipitating factors of suicide is conflict between the victim and someone very close to the victim [14]. Moreover, one study identified the dominant emotion at play in suicide in Micronesia as anger [15]. This suggests that adolescents who engage in conflict with another individual might be more likely to experience depressed mood and suicidal behaviors. Carrying a weapon was associated with suicide ideation for male adolescents and not for female adolescents. This finding is consistent with those indicating that male adolescents who carry a weapon and engage in physical fights in school and community settings are at increased risk for suicidal behaviors [30]. However, the variable that measures weapon carrying among adolescents does not determine whether the individual carried a weapon as a tool to cause violence or for self-protection as a result of feeling unsafe at school.

In this study, both female and male adolescents who experienced dating violence were more likely to experience suicide ideation, suicide plan, and suicide attempt. Studies have considered the effects of dating violence to occur exclusively among females on the assumption that female adolescents are more likely to experience severe forms of dating violence including sexual assault and physical injuries [31]. However, another study indicated that dating violence among adolescent relationships often involves the reciprocal use of violence by both partners [32]. Therefore, the effect of dating violence on depressed mood and suicidal behaviors should be further explored following a gender-inclusive approach, as both female and male adolescents might experience dating violence differently, and thus, it is crucial to consider their respective experiences [33]. Moreover, a study conducted in the US indicated that limited experience with romantic relationships causes most adolescents to have difficulty in dealing with and resolving problems they encounter in a relationship [34]. As a result, some may respond to relationship difficulties with violence while others may cope with self-inflicted violence, which can include suicidal behaviors.

In this study, there were significant gender differences in substance use behaviors associated with depressed mood, suicide ideation, suicide plan, and suicide attempt. Specifically, female adolescents reported more substance use behaviors involving “soft drugs” such as smoking, alcohol, and marijuana that were associated with depressed mood, suicide ideation, suicide plan, and suicide attempt. Male adolescents had more substance use behaviors involving “hard drugs” such as cocaine, heroin, and methamphetamine that were associated with depressed mood, suicide plan, and suicide attempt. The contrasting results for female and male adolescents’ associations between substance use behaviors, depressed mood, and suicidal behaviors might have been due to gender differences in the motivation for substance use [35]. One study investigating motivations for alcohol use among adolescents showed conformity to be more strongly related to alcohol use among male adolescents, whereas coping is more strongly related to alcohol use in female adolescents [36]. This could suggest that male adolescents are more likely to engage in “soft drugs” to socially connect with their peers, while female adolescents do so to avoid and cope with problems that may be associated with depressed mood and suicidal behaviors.

## Limitations

The present study is subject to several limitations. First, the questions related to mental health are limited. Questions on anxiety, post-traumatic stress disorder, or other past episodes related to mental health should be included to identify further psychological risk factors for depression and suicidal behaviors. Moreover, this study could not identify the impulsivity of suicidal behaviors among adolescents. A recent study suggested that a considerable number of suicide attempts occur due to sudden desires and were more common among younger people [37]. Second, all questions were self-reported, and the extent of under- or overreporting due to social desirability bias cannot be determined, although the survey questions demonstrated good test-retest reliability [22, 23]. Because the current study did not include adolescents who were absent from school on the day of the study and those whose parents did not provide permission for participation, the possibility of selection bias may be considerable. Due to this selection bias, adolescents who were at a higher risk of depressed mood, suicidal behaviors, and health risk behaviors may have been excluded from the study. Third, the current study followed a cross-sectional design and was unable to determine the causal relationship between health risk behaviors, depressed mood, and suicidal behaviors. The study also lacked a control group, and therefore could not determine other possible factors that might have affected the results. Fourth, the current study examined a number of combinations of independent and dependent variables, which might have yielded spurious statistical significance due to multiple comparisons. To counter this issue, we focused on our findings of associations of health risk behaviors that were consistently statistically significant across depressed mood, suicide ideation, suicide plan, and suicide attempt. Finally, survey questions related to the sociodemographic characteristics of adolescents were limited. During adolescence, social, environmental, situational, and cultural factors can significantly affect well-being [7, 10], and therefore, there could have been important confounders associated with depressed mood, suicidal behaviors, and health risk behaviors that were missed. Thus, residual confounding by variables missing from the questionnaire might have affected the associations found in the current study. Questions related to adolescents’ socioeconomic status, family structure, and peer involvement should be included to further investigate the factors associated with depressed mood and suicidal behaviors.

## Conclusion and public health implications

The prevalence of depressed mood and suicidal behaviors among high school adolescents was found to be higher in the CNMI than in the US mainland. Mental health among the indigenous population of the US Pacific territories should be further investigated to strengthen the monitoring of suicide and to enhance the quality of mental health data from the culturally distinct territories of the US. Moreover, when implementing public health interventions with limited resources, school health professionals might consider implementing and evaluating intervention programs for high school adolescents that concurrently address mental health issues such as depressed mood, suicide, and health risk behaviors. Specifically, the prevention of substance use, dating violence, bullying, and related topics could be mentioned while addressing depression and suicide. Furthermore, this study recommends implementing risk behavior and suicide prevention interventions separately for female and male adolescents, as they experience certain health risk behaviors, depressed mood, and suicidal behaviors differently.

## Data Availability

The data that support the findings of this study are available from the CDC upon request on their website. https://www.cdc.gov/healthyyouth/data/yrbs/contact.htm

## Abbreviations

ARR: Adjusted relative risk
CDC: Centers for Disease Control and Prevention
CI: Confidence interval
CNMI: Commonwealth of the Northern Mariana Islands
YRBS: Youth Risk Behavior Survey
